# Epidemiological investigation and pathogenicity analysis of waterfowl astroviruses in some areas of China

**DOI:** 10.3389/fmicb.2024.1375826

**Published:** 2024-03-11

**Authors:** Yang Li, Juan Luo, Jiajing Shang, Fuyou Zhang, Chunran Deng, Yingjie Feng, Ge Meng, Wenming Jiang, Xiaohui Yu, Hualei Liu

**Affiliations:** China Animal Health and Epidemiology Center, Qingdao, China

**Keywords:** waterfowl astrovirus, epidemiological investigation, genotype, phylogenetic analysis, pathogenicity

## Abstract

Waterfowl astroviruses are mainly duck astroviruses and goose astroviruses, of which duck astroviruses (DAstV-3, -4), goose astroviruses (GoAstV-1, -2) are the four new waterfowl 21 astroviruses in recent years, which can lead to enteritis, viral hepatitis, gout and reduce the growth performance of waterfowl, affecting the healthy development of the waterfowl farming industry. Since no targeted drugs or vaccines on the market, studies on the epidemiology of the virus are necessary for vaccine development. In this study, we collected 1546 waterfowl samples from 13 provinces in China for epidemiological investigation. The results showed that 260 samples (16.8%) were positive. Four species of astrovirus were detected in 13 provinces except Fujian province. Among the four sites tested, the highest positive rates were found in farms and slaughterhouses. Cross-host and mixed infection were observed in four species of waterfowl astroviruses. The whole genome of 17 isolates was sequenced and compared with published sequences. Genetic evolution and homology analysis showed that the isolated strains had high similarity to their reference sequences. To assess the pathogenicity of GoAstV, 7-day-old goslings were inoculated with GoAstV-1 and GoAstV-2 by the intramuscular route, and infected geese showed similar clinical signs, such as anorexia, depression, and weight loss. Organ damage was seen after infection, with histopathological changes in the heart, liver, spleen, kidney, and intestine, and higher viral loads in throat and anal swabs. These findings increase our understanding of the pathogenicity of GoAstV-1 and GoAstV-2 in goslings and provide more references for future research.

## 1 Introduction

Astroviruses are nonenveloped, single-stranded, positive-sense RNA viruses belonging to the Astrovirus genus, Astrovirus family. Their genomes are about 6.1 to 7.9 kb long and are organized in the following order: 5′ untranslated region (UTR), open reading frames (ORF1a, ORF1b, ORF2), 3′UTR and a poly (A) tail (An et al., 2020). ORF1a and ORF1b encode non-structural polyproteins (nsp1a and nsp1ab), ORF2 encoding capsid protein, which is a highly variable region of the genome and the main protein inducing host immune response in the immune response process (Méndez et al., 2007; Jamnikar-Ciglenecki et al., 2020).

The International Committee on Taxonomy of Viruses (ICTV) depending on the host infected by astroviruses classified them as two viral genera, including Mamastrovirus (mammal astrovirus, MAstV) and Astroviruses (avian astrovirus, AAstV) (An et al., 2020) Among them, MAstV mainly causes gastroenteritis and encephalitis in mammals, while AAstV mainly causes nephritis and enteritis in chickens, hepatitis in ducks, gout and nephritis in goslings (Chu et al., 2012; Zhang et al., 2018). Currently, at least 6 genetically distinct waterfowl astrovirus, including the Duck astrovirus (DAstV) 1, DAstV-2, DAstV-3, DAstV-4, Goose astrovirus (GoAstV) 1, GoAstV-2, have been identified based on the species of origin and viral genome characteristics (Strain et al., 2008; An et al., 2020). The DAstV-3, DAstV-4, GoAstV-1, GoAstV-2 are four new AAstVs in recent years, which have not been formally classified by ICTV, and cause duck hepatitis, goose gout and urate deposition, and the healthy development of China’s poultry farming industry poses a certain danger (Donato and Vijaykrishna, 2017). At the same time, the disease’s host diversity means there is a potential risk of intra−/cross-species transmission, and there is no commercial vaccine or targeted drug against the disease, making it difficult to prevent and control (Cortez et al., 2017; Kariithi et al., 2023). DAstV-3, DAstV-4, GoAstV-1 and GoAstV-2 are four waterfowl astroviruses reported since 2014, which can cause symptoms such as enteritis, hepatitis, urate deposition and gout, and have caused some economic losses to the healthy development of China’s waterfowl aquaculture industry since their discovery (Liao et al., 2015; Liu et al., 2016, 2022; Zhu and Sun, 2022). In particular, GoAstV is endemic in several provinces of China, where infection and mortality rates can reach 80 and 50% for infected geese and ducklings, respectively, and studies have detected the virus in infected ducks and chickens (Wei et al., 2020; Li et al., 2021). Study have shown that infection with GoAstV can revealed necrosis and abscission of renal tubular epithelial cells, inflammatory cell infiltration of the renal tissue, vacuolar degeneration of liver cells accompanied by inflammatory cell infiltration, disintegration and necrosis of spleen lymphocytes and encephalitis lesions have been observed in dead goslings (Yang et al., 2018; Zhang et al., 2018; Wang et al., 2020, 2021).

Therefore, investigation of the prevalence of waterfowl astrovirus and its continuous monitoring are very important for the prevention and control of this disease. In this study, we conducted an epidemiological survey of four waterfowl astroviruses on waterfowl samples from a total of 13 provinces in China to understand the prevalence and distribution of waterfowl astroviruses in China. We selected some positive samples for whole genome amplification, analysed the genetic evolutionary relationship. According to the epidemiological investigation, sequence analysis results and virus isolation, goose astrovirus was selected to carry out animal pathogenicity study in order to provide reference for the prevention and control of GoAstV.

## 2 Materials and methods

### 2.1 Ethics statement

The study’s protocol was conducted as per the animal welfare guidelines set by the World Organization for Animal Health and approved by the China Animal Health and Epidemiology Center (permit number: DWFL-2023-07).

### 2.2 Sample collection and processing

A total of 1,546 swab and tissue samples were collected from 13 provinces of China in 2022–2023 (the sample background information was shown in Supplementary Table S1). Swab samples were taken from both the faecal cavity and the oropharynx of the poultry and then mixed with sterile phosphate buffered saline (PBS, PH 7.4). Tissue samples were taken from the heart, liver and spleen of the diseased animals. The collected samples were mixed and homogenised with sterile phosphate buffered saline (PBS, pH 7.4) to a 20% suspension (w/v) using a High Throughput Tissue Homogeniser (Verder Shanghai Instruments and Equipment Co., Ltd.) and centrifuged at 10,000 rpm at 4°C for 5 min. The RNA was extracted with the 96-well nucleic acid extraction kit (Genfine biotech, Beijing, co., Ltd.) according to manufacturer’s instructions, and stored at −80°C.

### 2.3 Epidemiological investigation of four waterfowl astroviruses

Using the multiplex fluorescence quantitative PCR method established prior to this experiment with the RNA template extracted in 1.1, the 1,546 samples were assayed using the Evo M-MLV One Step RT-qPCR Kit II (Accurate Biotechnology, China). The real-time fluorescence quantitative RT-PCR amplification reaction system was: 10 μL 2× One step RT-qPCR Buffer II, 0.5 μL Pro Taq HS DNA Polymerase, 0.5 μL Evo M-MLV RTase Enzyme Mix II, the primers (10 μmol/L) of DAstV-3, DAstV-4, GoAstV-1 and GoAstV-2 are 0.8 μL, 0.8 μL, 0.6 μL and 0.6 μL respectively, and the probes (10 μmol/L) are 0.8 μL, 0.6 μL, 0.8 μL and 0.6 μL respectively, 2.0 μL of RNA template and RNase-free water were added to a final volume of 25 μL. The detection programme was as follows: 42°C for 5 min and 95°C 30 s, followed by 40 cycles of 95°C for 5 s and 58°C 30 s. Counted the number of positive samples for four waterfowl astroviruses in 13 provinces and analysed the distribution and positivity rates (Supplementary Table S2 for fluorescent primer probe information).

### 2.4 Virus genome-wide PCR amplification and analysis

With reference to the whole genome sequences of epidemic strains DAstV-3, DAstV-4, GoAstV-1 and GoAstV-2, PCR amplification primers were designed for their respective whole genome segmentation (Supplementary Table S3 for specific primer information). The primers were synthesised by Ruibo Xingke Biotechnology Co Ltd. (Qingdao, China). Some of the samples with positive results of multiplex fluorescence quantitative PCR were selected for whole genome amplification using the HiScript High Fidelity One step RT-PCR Kit (Vazyme Biotech, China). The PCR amplification products were detected by 1% agarose gel electrophoresis, and samples with positive nucleic acid amplification were sent to Sangon Biotech (Shanghai) Co, and the sequencing results were spliced using SeqMan software to obtain the whole genome sequence. The virulent strains from different hosts and different genotypes on GenBank were selected as reference sequences, and the evolutionary distances between the sequences and the reference sequences were calculated and the phylogenetic tree was constructed by (Neighbour-Joining) using Mega 7 software. Sequence homology was analysed using Megalign software (At present, there is only one reference strain for DAstV-3 and DAstV-4 on Genbank), therefore, subsequent genetic evolutionary analyses and homology analyses will only be performed with the respective reference strains (Supplementary Table S4 for reference primer sequence information).

### 2.5 Virus isolation and quantification

GoAstV-1 and GoAstV-2 isolation was attempted on samples that tested positive for GoAstV-1 and GoAstV-2 alone. The swab and tissue sample supernatants were centrifuged at 10,000 rpm for 5 min at 4°C to provide the inoculum for virus isolation. The supernatants were separated and filtered through a 0.22 μm filter, and the filtrate was inoculated into 10-day-old goose embryos (0.2 mL/egg) via the chorioallantoic membrane route. The goose embryos were placed in a fully automated incubator at 37°C, 55% humidity. Embryos that died beyond 24 h and those that survived until 10 days after inoculation were chilled to 4°C overnight.

According to the method described above, three generations of blind transmission are tested for the presence of GoAstV-1 or GoAstV-2 and other viruses, e.g., avian influenza virus (AIV), Newcastle disease virus (NDV), goose parvovirus (GPV), infectious bronchitis virus (IBV), avian reovirus (ARV), infectious bursal disease virus (IBDV), tembusu virus (TMUV).

Each dilution was inoculated with 5 each of 10-day-old goose embryos at a dose of 0.2 mL/embryo, placed in the incubator at 37°C for incubation, the embryos were photographed every 12 h, and the dead embryos within 24 h were discarded, embryos that died beyond 24 h and those that survived until 10 days after inoculation were chilled to 4°C overnight. The negatives and positives were determined by multiplex fluorescent quantitative PCR, according to the results of the test, the egg infectious dose (EID_50_) values were calculated according to the Reed-Muench method.

### 2.6 Source of goose embryos and experimental animals

The 1-day-old goose embryo was purchased from Shandong Haotai Experimental Animal Breeding Co, Ltd. The 1-day-old geese were purchased from Zhucheng Foreign Trade Co, Ltd. The geese were raised to 7 days of age for animal pathogenicity test. The experimental protocol was approved by the ethical committee for the China Animal Health and Epidemiology Center.

### 2.7 Pathogenicity assessment of GoAstV in goslings

To assess the pathogenicity of isolates, forty-five one-day-old healthy goslings were randomly divided into 3 groups (*n* = 15/group), of which 12 were infected with the virus and 3 were used as blank controls. The goslings in group A were infected with GoAstV-1 at a dose of 10^–4.67^/0.3 mL, the goslings in group B were infected with GoAstV-2 at a dose of 10^–3.83^/0.4 mL and the goslings in group C were infected with PBS as the negative control. The tissue with obvious pathological changes was fixed with 10% formaldehyde and histological examinations were performed with hematoxylin and eosin (HE) staining.

## 3 Results

### 3.1 Epidemiological survey of waterfowl astrovirus virus

**Figure 1:**
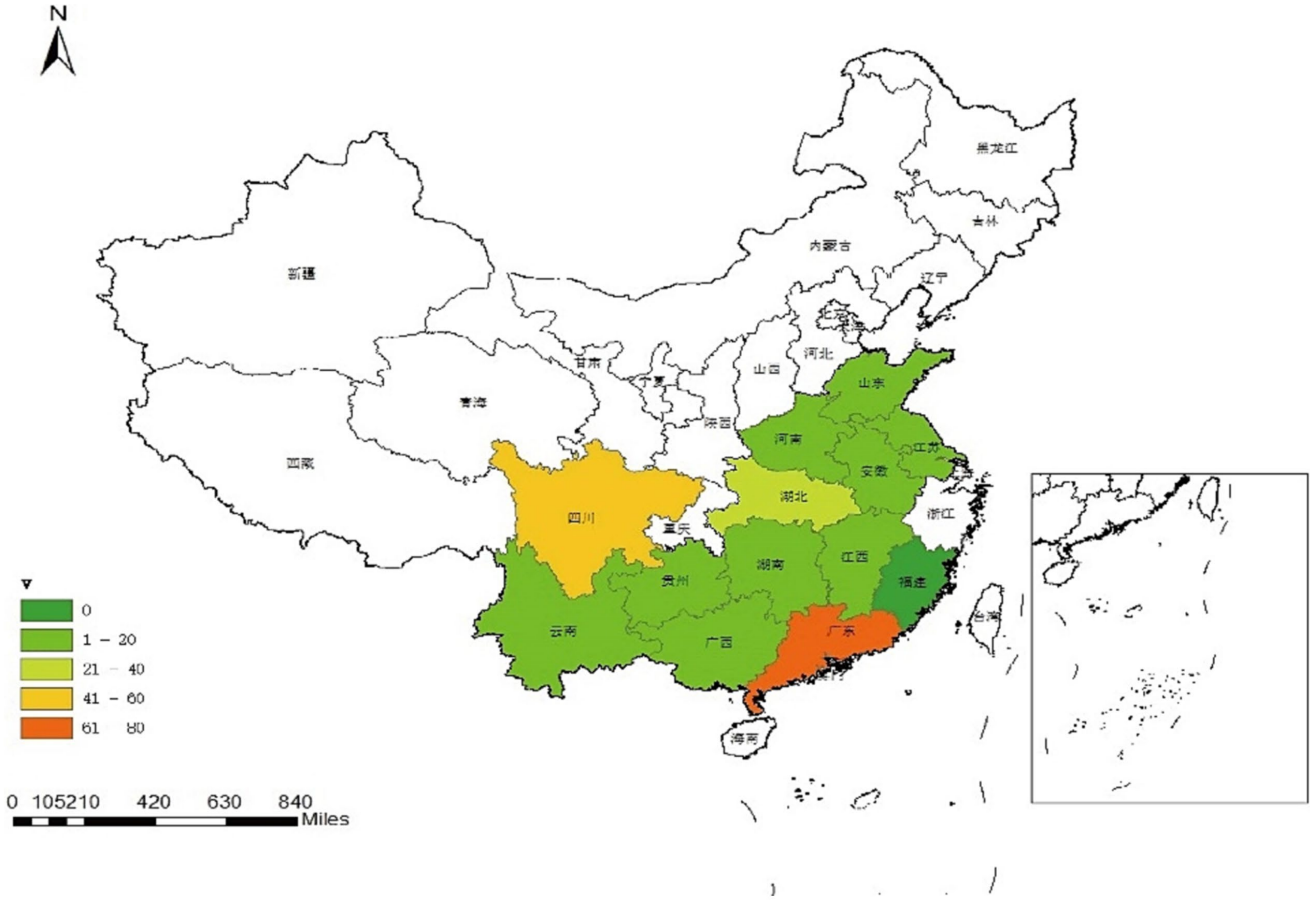
Geographic distribution map of waterfowl astrovirus positive samples. Map was created using ArcGIS by ESRI version 10.8 (http://www.esri.com). Map source data was obtained from the Natural Earth (http://www.naturalearthdata.com/).

The results of multiplex real-time fluorescence quantitative PCR for waterfowl samples are shown in Figure 1 and Table 1, with an overall positive rate of 16.8% (260/1546) for the samples examined. The positive rate of samples in provinces from 0–34.6%, with Guangdong province having the highest positivity rate of 34.6% and Fujian province having no positive samples. In the provinces of Guangdong, Guizhou, Hunan, Jiangsu, Jiangxi, Shandong and Sichuan, mixed infection with waterfowl astrovirus was detected.

**Table 1:** Statistical table of testing results of DAstV-3, DAstV-4, GoAstV-1 and GoAstV-2 in provinces.

Provinces	Sample number	DAstV-3	DAstV-4	GoAstV-1	GoAstV-2	Mixed infection	Positive rate
Anhui	134		2(1.5%)	3(2.2%)			5(3.7%)
Fujian	118						0(0%)
Guangdong	228	2(0.9%)	63(27.6%)	8(3.5%)		6(2.6%)	79(34.6%)
Guangxi	186	2(1.1%)	17(9.1%)				19(10.2%)
Guizhou	74		19(25.7%)			1(1.4%)	20(27.0%)
Henan	58		1(1.7%)				1(1.7%)
Hubei	130		33(25.4%)				33(25.4%)
Hunan	125		11(8.8%)			1(0.8%)	12(9.6%)
Jiangsu	58			1(1.7%)	1(1.7%)	1(1.7%)	3(5.2%)
Jiangxi	125	5(4.0%)	1(0.8%)	3(2.4%)		1(0.8%)	10(8.0%)
Shandong	96	10(10.4%)	1(1.0%)		6(6.3%)	3(3.1%)	20(20.8%)
Sichuan	162	8(4.9%)	24(14.8%)	9(5.6%)		13(8.0%)	54(33.3%)
Yunan	52		4(7.7%)				4(7.7%)
Total	1,546	27(1.7%)	176(11.4%)	24(1.6%)	7(0.5%)	26(1.7%)	260(16.8%)

The 1,546 samples tested were from 125 sites, including 53 retail markets, 33 wholesale markets, 6 slaughterhouses and 33 poultry farms, as shown in Table 2. Host species-specific AAstV infection were identified in numerous samples collected at all the four types of poultry sites. Poultry farms and slaughterhouses had the highest positive rates, followed by wholesale markets.

**Table 2:** Test results of DAstV-3, DAstV-4, GoAstV-1, and GoAstV-2 in samples from different sites.

Type of site	Site positive number	Positive rate
Retail market (*n* = 53)	14	26.4%
Wholesale market (*n* = 33)	20	60.6%
Slaughterhouses (*n* = 6)	4	66.7%
Poultry farms (*n* = 33)	22	66.7%
Total (*n* = 125)	60	48%

The positive rate of goose samples was higher than that of duck samples. Meanwhile, cross-host infection was present for DAstV-3, DAstV-4, GoAstV-1 and GoAstV-2, with DAstV-4 being the most severe cross-host phenomenon with a positive rate of 54.2% (141/260). There were 26 mixed infection samples in the sample, the overall mixed infection rate was 10% (26/260). The samples of mixed infection were double mixed infection and triple mixed infection, and the number of double mixed infection was more (Table 3).

**Table 3:** Detection results of DAstV-3, DAstV-4, GoAstV-1 and GoAstV-2 in samples from different hosts.

	Duck	Goose	Positive rate (positive samples/total)
DAstV-3	23	4	1.7% (27/1546)
DAstV-4	35	141	11.3% (176/1546)
GoAstV-1	4	20	1.6% (24/1546)
GoAstV-2	0	7	0.5% (7/1546)
DAstV-3 + DAstV-4	2	1	0.2% (3/1546)
DAstV-3 + GoAstV-1	1	7	0.5% (8/1546)
DAstV-4 + GoAstV-1	0	8	0.6% (9/1546)
DAstV-4 + GoAstV-2	1	1	0.06% (1/1546)
GoAstV-1 + GoAstV-2	0	4	0.3% (4/1546)
DAstV-4 + GoAstV-1 + GoAstV-2	1	0	0.06% (1/1546)
Positive rate	4.3% (67/1546)	12.5% (193/1546)	16.8% (260/1546)

### 3.2 Genetic characterisation of waterfowl astrovirus

**Figure 2:**
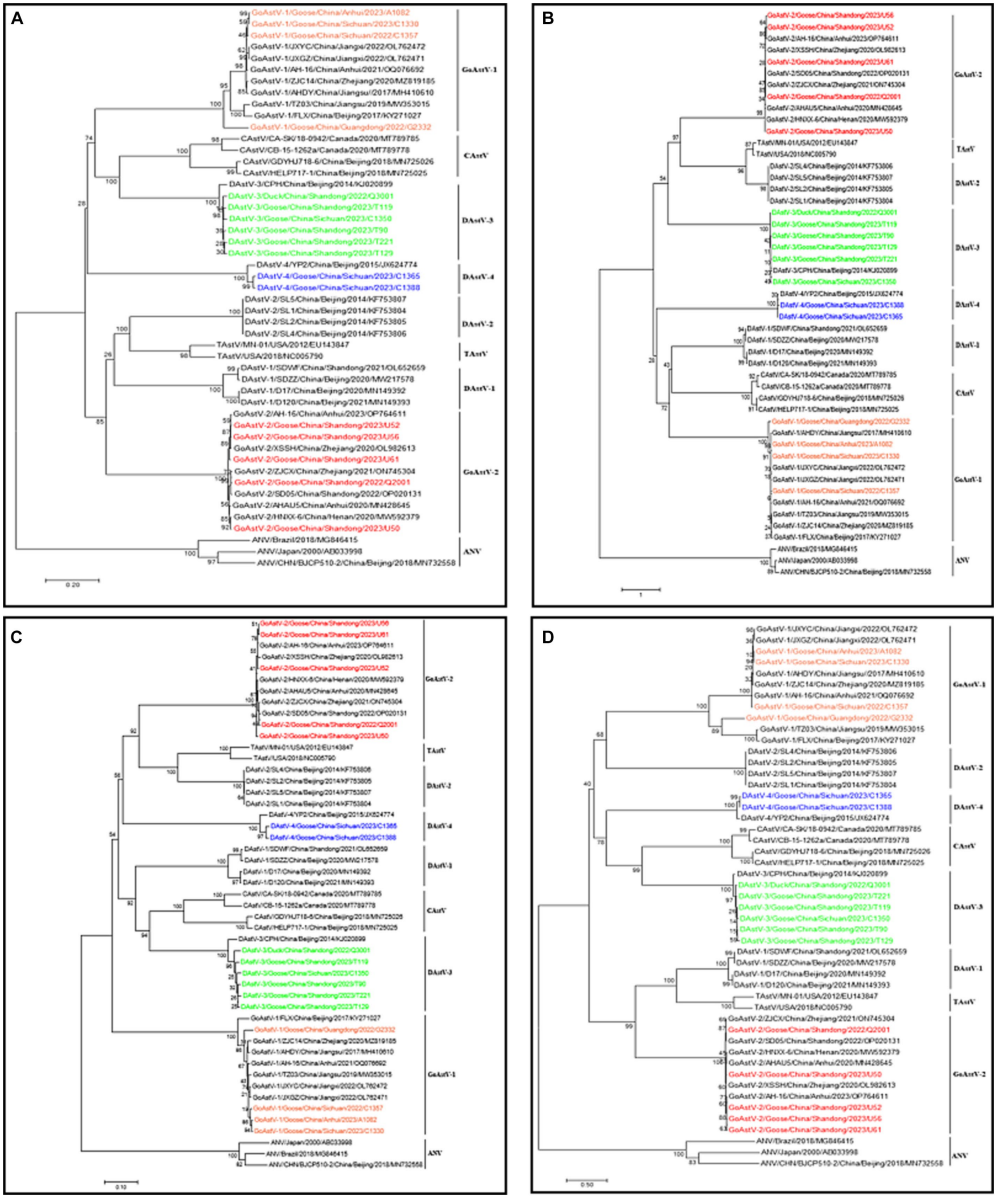
Phylogenetic tree of waterfowl astrovirus **(A)** Whole genome **(B)** ORF1a **(C)** ORF1b **(D)** ORF2. Different isolates are represented with distinct colors. Red represents GoAstV-2 isolates; green represents DAstV-3 isolates; orange represents GoAstV-1 isolates; blue represents DAstV-4 isolates.

Some of the positive samples were selected for whole genome amplification of the virus and the whole genome sequence of 17 strains was successfully amplified (Table 4). In order to further evaluate the evolutionary relationship between the sequenced columns and other reference sequences, a genetic evolutionary analysis tree was constructed using Mega 7 software. The whole genome sequence, ORF1a, ORF1b and ORF2 genes all showed that the six full-length sequences of DAstV-3, two full-length sequences of DAstV-4, four full-length sequences of GoAstV-1, and five full-length sequences of GoAstV-2 obtained in this study all belong to the same branch as their respective reference strains (Figure 2).

**Table 4:** The genome length of the waterfowl astrovirus strain obtained in this study.

GenBank number	Strains	Type	GenBank number	Strains	Type
OR907125	T221	DAstV-3	OR907132	A1082	GoAstV-1
OR907126	T90	DAstV-3	OR907133	C1357	GoAstV-1
OR907127	C1350	DAstV-3	OR907134	G2332	GoAstV-1
OR907128	T129	DAstV-3	OR902761	Q2001	GoAstV-2
OR907129	T119	DAstV-3	OR902762	U61	GoAstV-2
OR907130	Q3001	DAstV-3	OR902763	U56	GoAstV-2
OR902759	C1388	DAstV-4	OR902764	U52	GoAstV-2
OR902760	C1365	DAstV-4	OR902765	U50	GoAstV-2
OR907131	C1330	GoAstV-1			

Genome-wide sequence homology analysis showed that the homology between 6 DAstV-3 isolates and DAstV-3 reference sequences was 96.2–96.7%, between 2 DAstV-4 isolates and DAstV-4 reference sequences 96.2–96.3%, between 4 GoAstV-1 isolates and GoAstV-1 reference sequences 86.5–98.8%, and between 5 GoAstV-2 isolates and GoAstV-2 reference sequences 96.3–99.2%. The homology between GoAstV-2 and 6 reference sequences was 96.3–99.2%.

### 3.3 Replication kinetics and histopathology of GoAstV infected goslings

#### 3.3.1 Clinical symptoms

Goose infected with GoAstV-1 at 4 dpi showed symptoms of white loose stools, depression, bunching and reduced feed intake, death began at 3 dpi and the mortality rate was 33% at 15 dpi, with a few individuals developing drowsiness, convulsions and opisthotonus before death. Goose infected with GoAstV-2 at 1 dpi, depression, bunching, reduced feed intake, and mortality started at 1 dpi and was 41.6% by 15 dpi. Meanwhile, no significant clinical signs or death were observed in the PBS control group. In this animal experiment, the weight of each gosling was determined at 5, 10 and 15 dpi. The results showed that the infected group had a remarkably lower weight than the uninfected group, which indicated that GoAstV-1 and GoAstV-2 significantly influenced the growth of goslings.

#### 3.3.2 Autopsy lesion

**Figure 3:**
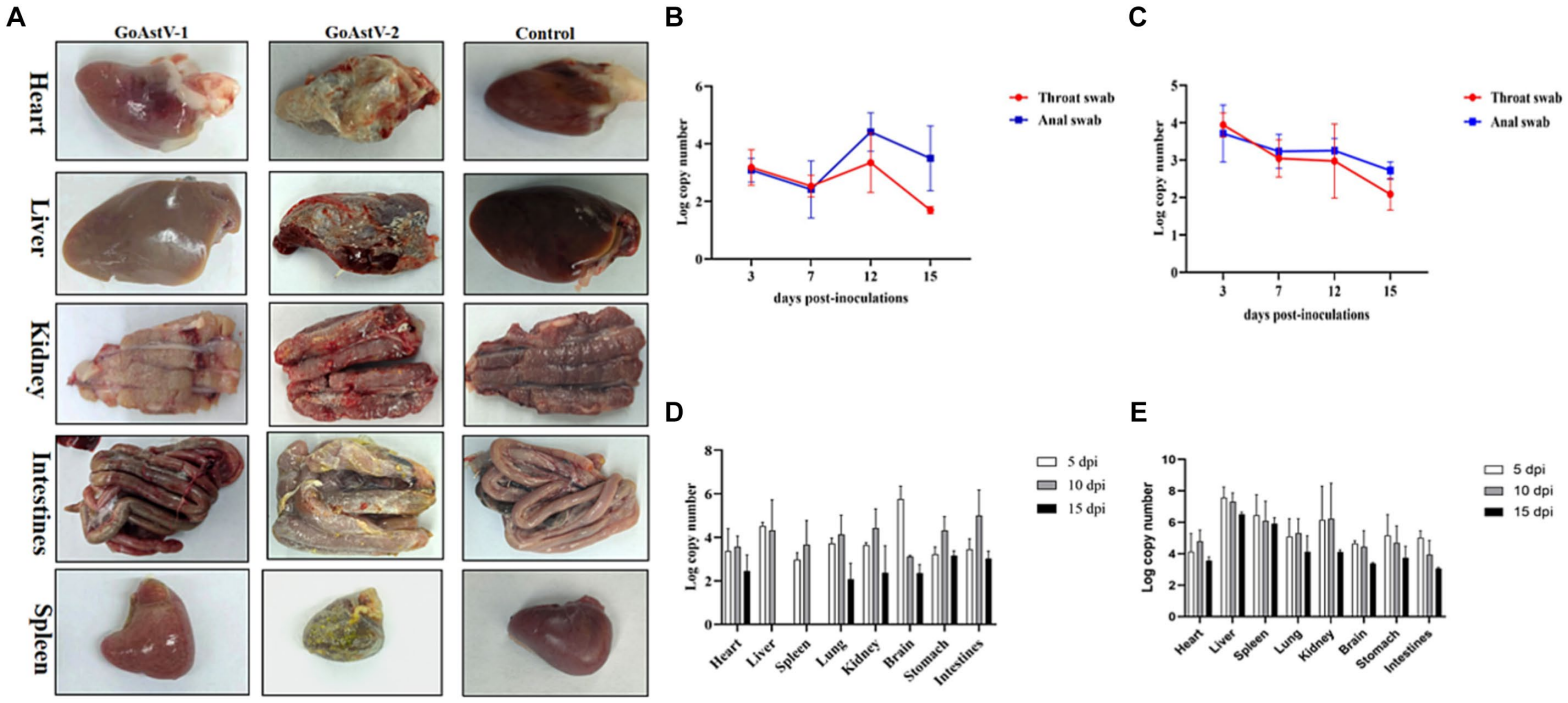
**(A)** Clinical necropsy lesions in GoAstV-1 and GoAstV-2 infected geese. **(B)** The viral copy numbers in throat and anal swabs of goslings infected with GoAstV-1. **(C)** The viral copy numbers in throat and anal swabs of goslings infected with GoAstV-2. **(D)** The viral copy numbers in tissue samples of GoAstV-1 infection group. **(E)** The viral copies in tissue samples of GoAstV-2 infection group.

At the autopsy of these goslings, there was obvious congestion on the surface of the heart after GoAstV-1 infection. There were haemorrhages on the surface of the liver. The kidneys were pale with obvious striated haemorrhages, and one side of the ureter showed a white line. The intestines were swollen with partial haemorrhage; there were needle-like haemorrhages on the surface of the spleen. The clinical manifestations of GoAstV-2 infection were obvious deposition of urate on the surface of the heart, liver, kidney, spleen and intestine, and swelling of the kidney and intestine (Figure 3A).

#### 3.3.3 Viral load in throat and anal swabs

Virus was detected in throat and anal swabs after GoAstV-1 and GoAstV-2 infection. The highest viral load was reached at 12 dpi in the GoAstV-1 infected group and at 3 dpi in the GoAstV-2 infected group. In addition, the viral load of both challenge groups started to decrease after 12 dpi (Figures 3B,C).

#### 3.3.4 Viral load in tissues and organs

To explore virus replication and distribution in goose, heart, liver, spleen, lung, kidney, brain, stomach, and intestine samples were collected at 5 dpi, 10 dpi, and 15 dpi after infection for detection. Both GoAstV-1 and GoAstV-2 have extensive tissue tropism after infection and the viruses can be detected in many tissues and organs. After GoAstV-1 infection, the virus in tissues and organs could be detected at 5 dpi, and the viral load in brain tissues was the highest. The viral load tended to be level at 10 dpi, and no virus was detected in liver and spleen tissues at 15 dpi (Figure 3D). After GoAstV-2 infection, the virus contented in the liver was the highest at 5 dpi and 10 dpi, and the virus contented in the heart was the lowest. At 15 dpi, the virus contented in the liver was the highest, and the virus contented in the enterovirus was the lowest. The results showed that the viral load in liver, kidney and spleen was high (Figure 3E).

#### 3.3.5 Histopathology in different tissues

**Figure 4:**
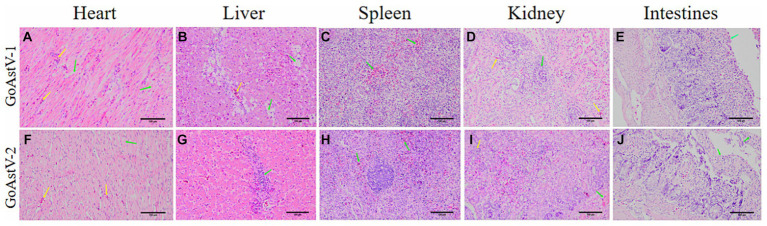
Histopathological changes in goslings, experimentally infected with GoAstV. **(A)** Myocardial cells were swollen and broken (green arrow), and a small amount of red blood cells appeared in the myocardial fibrous interstitium (yellow arrow). **(B)** Moderate steatosis (green arrow) around the hepatic sinusoid space and central veins (yellow arrow). **(C)** There were diffuse red cells in the splenic pulp (green arrow). **(D)** Renal tubular epithelial cells atrophy and degeneration (green arrow), and a small number of red blood cells appeared in the interstitium (yellow arrow). **(E)** Intestinal villi were broken and shed (green arrow). **(F)** A small number of cardiomyocytes were swollen and the gap widened (green arrow). There were a few red cells in the stroma (yellow arrow). **(G)** Moderate inflammatory cell infiltration in the hepatic sinusoidal space and around the central vein (green arrow). **(H)** A few diffuse red blood cells were found in the splenic pulp (green arrow). **(I)** Degeneration and necrosis of renal tubular epithelial cells (green arrow), atrophy and shrinkage of glomeruli (yellow arrow). **(J)** Intestinal villi shed and epithelial cells disappeared (green arrows). HE-stained, Magnification, 20 × 10.

In addition, histopathological analysis of HE staining was used to assess the degree of histopathological injury. Swelling and rupture of cardiomyocytes were observed after GoAstV-1 infection. There was moderate steatosis in the hepatic sinusoidal space and central veins. The splenic pulp was indistinct with diffuse hemorrhage. Some renal tubular epithelial cells were atrophic and degenerated. After GoAstV-2 infection, the myocardial cells were disorderly arranged, swollen and fractured. There was inflammatory cell infiltration in the hepatic sinusoidal space and central vein. There were a few diffuse red blood cells in the splenic pulp. Renal interstitial inflammatory cell infiltration, glomerular atrophy, renal tubular epithelial cells degeneration, necrosis; Intestinal villi were arranged disorderly and epithelial cells disappeared (Figure 4).

## 4 Discussion

There are many types of waterfowl astroviruses, among which DAstV-3, DAstV-4, GoAstV-1 and GoAstV-2 are four newly discovered avian astroviruses in recent years, mainly causing duck hepatitis, goose gout, urate deposition or enteritis. Since the report of DAstV-4, there have been few studies on this virus, and its clinical manifestations and pathogenicity need to be further studied. In order to further study and understand the distribution of astroviruses in waterfowl, we conducted an epidemiological survey to obtain the infection status of astroviruses in waterfowl in some areas of China, and summarized the geographical distribution, host distribution, prevention and control of these pathogens.

The positive rates of individuals in 13 provinces surveyed in this study ranged from 0–34.6%. Except for Fujian province, positive samples were detected in the other 12 provinces, indicating that the infection rate of these four waterfowl astroviruses was high and widely distributed. Due to the uneven number of samples detected in different provinces, the number of positive samples also varied. The rate of individual DAstV-4 positive cases in goose exceeded that in ducks. Additionally, a substantial number of geese tested positive for DAstV-4 infection, whereas mixed infections with diverse combinations were identified in both ducks and geese. This indicates potential cross-host transmission between duck astrovirus and goose astrovirus, as well as concurrent waterfowl astrovirus infections within a single host. The positive rate of DAstV-4 in 4 waterfowl astroviruses was higher than that of the other three detected viruses. There were infections in poultry farms, slaughterhouses, wholesale markets and retail markets, the results of epidemiological testing by Zhang et al. (2022a). Therefore, it is necessary to strengthen the management and immunization program of poultry farms to block the spread of the disease from the source, and strengthen the surveillance of the astrovirus from waterfowl in these 4 links.

For the positive samples detected by multiplex real-time RT-PCR, part of the samples were sequenced by using the primers specific for their whole genome, and a total of 17 full-length sequences were detected, including 6 DAstV-3 strains, 2 DAstV-4 strains, 4 GoAstV-1 strains and 5 GoAstV-2 strains.

The GoAstV include GoAstV-1 and GoAstV-2, of which GoAstV-2 is a new type of goose-origin astrovirus discovered in 2016, sharing only 30.0–50.5% homology with the previously discovered GoAstV (Yuan et al., 2018). In 2019, an outbreak of highly acute infectious disease characterized by visceral gout in ducklings occurred in Shandong Province with a mortality rate of 30% (Chen et al., 2020). The causative agent was identified as GoAstV-2. Subsequently, a similar outbreak with a mortality rate of 61% was reported in Henan Province in 2020. Whole genome sequence analysis revealed that the host range of GoAstV is expanding and may have potential implications for other poultry species (Chen et al., 2021). In recent years, the clinical cases of GoAstV infection have been increasing and reported in different provinces of China, which has brought huge economic losses to poultry industry. In this study, the epidemiologically positive samples were separated and the pathogens GoAstV-1 and GoAstV-2 were successfully isolated. The pathogens GoAstV-1 and GoAstV-2 were isolated, which can reproduce well in goose embryos. The pathogenicity of GoAstV was systematically investigated in a goose model. In the stage after GoAstV-1 and GoAstV-2 infection, geese showed clinical symptoms such as loose white stool, depression, clumps, and reduced feed intake. During infection, the virus can be detected in throat swabs and anal swabs. Therefore, in the clinical surveillance of waterfowl astrovirus and in the investigation of post-onset infection, throat swabs and anal swabs can be collected for detection. Autopsy following GoAstV-1 infection indicated bleeding in vital organs such as the heart, liver, spleen, kidney and intestine. These findings align with Zhang’s study (Zhang et al., 2022a). The viral load in brain tissue reached the peak earliest. The viral load levels in kidney and intestine were stable at each time point. The results of liver and spleen were negative at 15 dpi. Renal tubular epithelial-interstitial hemorrhage and diffuse red blood cells in the spleen, consistent with previous studies (Zhang et al., 2022b).

Necropsy after GoAstV-2 infection showed obvious urate deposition and obvious lesions in various tissues and organs. The viral load in liver was the highest, followed by kidney and spleen, which was consistent with the results of Tian research (He et al., 2023). Previous research has indicated that viral loads within the kidney are higher compared to the liver, heart, and spleen (Yin et al., 2021). Simultaneously, GoAstV-2 infection can lead to substantial harm to the liver and kidneys, augment the production of uric acid, impede its excretion, and elevate serum uric acid levels. The rate of formation of renal urate is greater than the ability of urinary organs to excrete it, which in turn causes gout. Uric acid accumulates in the blood and can be transferred to any organ of the body through the blood circulation, resulting in urate deposits, such as the heart, liver, and kidneys, where gout lesions are more pronounced (Zhu and Sun, 2022). Furthermore, the histopathological observations revealed that the epithelial cells of the renal tubules in infected geese underwent necrosis and degeneration, aligning with earlier research findings (Zhu et al., 2022).

In conclusion, there are some differences in the tissue damage vary of the two goose astroviruses. The GoAstV-1 infection after each organs have obvious symptoms of hemorrhage brain viral load in the highest. There was moderate steatosis in the hepatic sinusoidal space and central veins. The GoAstV-2 showed obvious urate deposition in all tissues and organs, and high viral load in liver. There was inflammatory cell infiltration in the hepatic sinusoidal space and central vein.

## 5 Conclusion

In this study, 4 species of astroviruses were detected in waterfowl. The results showed that there were different degrees of infection in waterfowl astroviruses in 13 provinces, except Fujian Province. The positive rate of DAstV-4 was high, goose astrovirus infection was detected in ducks, duck astrovirus infection was detected in goose, and different combinations of duck astrovirus and goose astrovirus co-infection were detected, indicating that these four waterfowl astroviruses may have cross-host transmission and the infection of different astroviruses in the same host, but the specific pathogenic mechanism and influence need to be further studied. The positive rates in livestock farms and slaughterhouses were high. It is necessary to strengthen the feeding management and immunization program in livestock farms to stop the transmission of the pathogen from the source. The positive samples were amplified and 17 whole genome sequences were successfully amplified. Genetic evolution and homology analyses showed that the 17 whole genome sequences were 6 DAstV-3, 2 DAstV-4, 4 GoAstV-1 and 5 GoAstV-2. To explore the pathogenicity of GoAstV-1 and GoAstV-2 to geese, respectively, and seven-day-old geese were infected by intramuscular injection. The results showed that both GoAstV-1 and GoAstV-2 infection affected the growth rate of geese, and the infection had a wide tissue tendency, resulting in a certain degree of tissue damage.

## Data availability statement

The datasets presented in this study can be found in online repositories. The names of the repository/repositories and accession number(s) can be found in the article/Supplementary material.

## Ethics statement

The animal study was approved by the World Organization for Animal Health and approved by the China Animal Health and Epidemiology Center. The study was conducted in accordance with the local legislation and institutional requirements.

## Author contributions

YL: Conceptualization, Writing – original draft, Writing – review & editing. JL: Methodology, Validation, Writing – original draft, Writing – review & editing. JS: Conceptualization, Investigation, Writing – review & editing. FZ: Data curation, Software, Writing – review & editing. CD: Formal analysis, Methodology, Writing – review & editing. YF: Investigation, Validation, Writing – review & editing. GM: Investigation, Validation, Writing – review & editing. WJ: Supervision, Validation, Writing – review & editing. XY: Supervision, Validation, Writing – review & editing. HL: Funding acquisition, Project administration, Writing – original draft, Writing – review & editing.
