# Supplementary material for: Epidemiological investigation and pathogenicity analysis of waterfowl astroviruses in some areas of China
Source: Front Microbiol. 2024 Mar 11;15:1375826. doi: 10.3389/fmicb.2024.1375826 (PMC10961457; doi:10.3389/fmicb.2024.1375826)
Supplement: Supplementary file 1 [file Table_1.docx]

Supplementary Table 1. Background information of the samples

| **Province** | **Host** | **Number of swab samples** | **Number of tissue samples** |
| --- | --- | --- | --- |
| Guangdong | duck, goose | 227 | 1 |
| Guangxi | duck, goose | 185 | 1 |
| Shandong | duck, goose | 83 | 13 |
| Sichuan | duck, goose | 162 | 0 |
| Hubei | duck, goose | 130 | 0 |
| Hunan | duck, goose | 125 | 0 |
| Jiangsu | duck, goose | 55 | 3 |
| Jiangxi | duck, goose | 124 | 1 |
| Henan | duck, goose | 58 | 0 |
| Fujian | duck | 118 | 0 |
| Anhui | duck, goose | 134 | 0 |
| Guizhou | duck, goose | 74 | 0 |
| Yunnan | duck, goose | 52 | 0 |

Supplementary Table 2 Multiplex fluorescence quantitative PCR primer probe information

| **Primer probes** | **Sequences（5′—3′）** |
| --- | --- |
| DAstV-3-F | TGCGTACACCAACTAGAAAACG |
| DAstV-3-R | TAGCAGCATACTCCTCAAC |
| DAstV-3-P | FAM-ATGCCAGCGTTCGTGAGTATCT-BHQ1 |
| DAstV-4-F | GATCATCGAGAAGGCCCTTATGG |
| DAstV-4-R | TGTTGTTTCGCTACCCACCTCTC |
| DAstV-4-P | VIC-GCTCCACACAACCGCTCGTCC-BHQ1 |
| GoAstV-1-F | TCAACGTTTGAGTTTGGGTACTT |
| GoAstV-1-R | AACAGATCGGCATACATCTTTA |
| GoAstV-1-P | ROX-CTTAAGTATATTTGCTATGGAGATGACACGTT-BHQ2 |
| GoAstV-2-F | GAGTGGACAACAATATGTGTAA |
| GoAstV-2-R | CATCATCGCCATAGCAAATGA |
| GoAstV-2-P | CY5-GTCATTGCCGACGCTAAGATTA-BHQ2 |

Supplementary Table 3 Genome-wide amplification primer sequences of four waterfowl astroviruses

| **Primer name** | **Sequence (5′—3′)** |
| --- | --- |
| DAstV-3 Whole Genome Amplification Primers | |
| DAstV-3 F1 | CAAGCGAGTGGAGAGGTTTCA |
| DAstV-3 R1 | GCTCGGTTGTATGAGTTGGAA |
| DAstV-3 F2 | CAGCTGGGTGGAGAAATATCA |
| DAstV-3 R2 | GTGCTTCAATTTCCCGATCTA |
| DAstV-3 F3 | AGCTGGCAGTGAATGGGCAAC |
| DAstV-3 R3 | CCAATCAGGTCGACATTATCT |
| DAstV-3 F4 | TGAGTGTCCACGTTGTAAGCA |
| DAstV-3 R4 | GCCATAATGCTACTTTCGGTC |
| DAstV-3 F5 | ACGTCTGGCTTACTAACTTTG |
| DAstV-3 R5 | CACCACCAGTTTGATTGCTCG |
| DAstV-3 F6 | ACTTAGTTGGCCCACGTTCAG |
| DAstV-3 R6 | AAGGCCTTCTCCATCATCAGC |
| DAstV-4 Whole Genome Amplification Primers | |
| DAstV-4 F1 | CGTCGACAAGCTAGGGTGGAA |
| DAstV-4 R1 | ACCAGATTAAGAATGCTACGG |
| DAstV-4 F2 | TGCCACTCCTATTTGTCTGCT |
| DAstV-4 R2 | GTGAGATCTTTGTTGCCTCTA |
| DAstV-4 F3 | GCTGGTCATGTGGTTAGGGAT |
| DAstV-4 R3 | GCTTGTTCTCTAAGTCCATCG |
| DAstV-4 F4 | ACCGGTAGTTCAACAGTGTGC |
| DAstV-4 R4 | CCATAGAAGTCAACCTCATGT |
| DAstV-4 F5 | TGAAGAATTGGAGAATCATGTCAT |
| DAstV-4 R5 | TCTTATTTCGGTCCTCCCTACCA |
| DAstV-4 F6 | ATTGGCTTGTCTTTCTGTGG |
| DAstV-4 R6 | TGATTTGTGCACCTGTTGTTC |
| DAstV-4 F7 | GCTACTGATACACTCGGGCCA |
| DAstV-4 R7 | GGTGATTCCCGTTGCACTAGA |
| GoAstV-1 Whole Genome Amplification Primers | |
| GoAstV-1 F1 | CCGAAAGCGTTGGTGAGAGG |
| GoAstV-1 R1 | AGATGTTAACCGCAACTGTGAG |
| GoAstV-1 F2 | TGAAGCCCTTGAGCAGAGTAG |
| GoAstV-1 R2 | CTTCAACACCATCTTCATCTA |
| GoAstV-1 F3 | TTATGATGATTTCTGGCTGATGCAA |
| GoAstV-1 R3 | AGGCAGGTGTGGATTTAACATT |
| GoAstV-1F4 | AGATCCACTCCTGGGTATTGT |
| GoAstV-1 R4 | AACACCACCACAAAGGTCG |
| GoAstV-1 F5 | CAATGAGTGTGGATGGGGTGAGTAC |
| GoAstV-1 R5 | GTCCTTCCTCGTGCCTTAGCCT |
| GoAstV-1 F6 | GAGAATGTAAAGGTGCAGATGG |
| GoAstV-1 R6 | CTTCCTTATAATCCACCAGCCA |
| GoAstV-1 F7 | GTAGAGGCCAATGTCAGGTAT |
| GoAstV-1 R7 | GTTAGTCACCTTGTCCACCCT |
| GoAstV-1 F8 | GTAGTCATGGAAGTGCAAGATCCG |
| GoAstV-1 R8 | AAACAGTGAGAGAACACAAGGT |
| GoAstV-2 Whole Genome Amplification Primers | |
| GoAstV-2 F1 | CGTGAGGAAAGGAAGACTGAT |
| GoAstV-2 R1 | TGAACCGGAATCCAACCCCTG |
| GoAstV-2 F2 | AAGATGGTGAGATTTGGGCAG |
| GoAstV-2 R2 | AGCATATTCATCTTGTTGCCA |
| GoAstV-2 F3 | GTTCAAGAGTGTAGAGGAGCT |
| GoAstV-2 R3 | ACACTATTGGGTGCATTTTCG |
| GoAstV-2F4 | CATTGCCGACGCTCAGATTAC |
| GoAstV-2 R4 | ACCAGAATTTGAAGCAGCACC |
| GoAstV-2 F5 | GGCACCACAAGTTCCCTATAC |
| GoAstV-2 R5 | AAGCCTAATGAGAAGGTGCAG |

Supplementary Table 4 Reference strain sequence information downloaded from Genbank

| Reference Sequences | Place of separation/year | genotypes | Reference Sequences | Place of separation/year | genotypes |
| --- | --- | --- | --- | --- | --- |
| EU143847 | USA/2012 | TAstV | OL652659 | China/2021 | DAstV-1 |
| NC005790 | USA/2018 | TAstV | MN149392 | China/2020 | DAstV-1 |
| OQ076692 | China/2021 | GoAstV-1 | MN149393 | China/2021 | DAstV-1 |
| OL762472 | China/2022 | GoAstV-1 | KF753807 | China/2014 | DAstV-2 |
| OL762471 | China/2022 | GoAstV-1 | KF753806 | China/2014 | DAstV-2 |
| MZ819185 | China/2020 | GoAstV-1 | KF753805 | China/2014 | DAstV-2 |
| MW353015 | China/2019 | GoAstV-1 | KF753804 | China/2014 | DAstV-2 |
| KY271027 | China/2017 | GoAstV-1 | KJ020899 | China/2014 | DAstV-3 |
| MH410610 | China/2017 | GoAstV-1 | JX624774 | China/2015 | DAstV-4 |
| OP764611 | China/2023 | GoAstV-2 | MG846415 | Brazil/2018 | ANV |
| OP020131 | China/2022 | GoAstV-2 | MN732558 | China/2018 | ANV |
| OL982613 | China/2020 | GoAstV-2 | AB033998 | Japan/2000 | ANV |
| MW592379 | China/2020 | GoAstV-2 | MT789778 | Canada/2020 | CAstV |
| MN428645 | China/2020 | GoAstV-2 | MT789785 | Canada/2020 | CAstV |
| ON745304 | China/2021 | GoAstV-2 | MN725026 | China/2018 | CAstV |
| MW217578 | China /2020 | DAstV-1 | MN725025 | China/2018 | CAstV |
